# Address on English Recollections of a German Surgeon

**Published:** 1872-10

**Authors:** 

**Affiliations:** Hanover


*An Address on English Recollections of a German Surgeon.* Being a Speech delivered at St. Thomas's Hospital, May 23, 1872. By Dr. Stromeyer, of Hanover.

Gentlemen—I suppose I may leave it to the kind care of my youngest English friend, Mr. William McCormac, to account for the liberty I take in addressing you. Let me ask your indulgence for spoiling the Queen's English, which is not my native language. This is the first time that I speak to an English audience. As a surgeon, I dare say, I am not quite a foreigner, having got a sprinkling of English surgery even by inheritance. My father, who was a member of the Royal Medico-Chirurgical Society in London, and well known in his time, by having introduced vaccination in Germany, was a regular pupil of St. Thomas's Hospital, from 1792 to 1793, under Mr. Cline, at a time when Sir Astley Cooper was a demonstrator of anatomy there. He had a very high opinion of English surgery, and used to say that the best surgeons in the universe were to be found in London; that during a twelve months' presence there he witnessed only a very few cases in which his opinion was different about the propriety of the operations which he daily saw performed. He was able to judge for himself, being already thirty years old when in London, and having been a pupil and assistant of Professor Richter, in Gottingen. I followed my father's example, and have been a pupil myself at St. Thomas' Hospital in 1827 and 1828. Mr. Henry Green introduced me there, and made me acquainted with the splendid circle of surgeons then living in London: Benjamin Travers and John Tyrrell, of St. Thomas's Hospital; Bransby Cooper, Aston Key, and Mr. Morgan, of Guy's Hospital; William Lawrence and Henry Erle, of St. Bartholomew's Hospital; Sir Benjamin Brodie and Mr. Rose, of St. George's Hospital; Sir Charles Bell, of Middlesex Hospital; Mr. Guthrie, of Westminster Hospital; Mr. Wardrop, of Westminster Eye Infirmary. Sir Astley Cooper had already retired to the country. I have only seen him on an occasional visit to St. Thomas' Hospital where he used to come from time to time when he was tired, as he said, of looking after the ewes. It was highly gratifying to see how his presence used to be hailed. The same scene took place when old John Abernethy appeared in St. Bartholomew's Hospital. The students flocked around him, and he generally gave them a speech, in parting, in the open court of the Hospital, ending by quoting Shakspeare. Being very partial myself to the great poet, I liked these quotations, which reminded me of Sydenham recommending to read "Don Quixote." For a surgeon, nothing is so injurious as dullness; he must always be in good spirits when his services are required. Sir Astley Cooper used to say that a surgeon ought not to read too much; but this, I suppose, meant dull authors, not Shakespeare or Cervantes, who are both of them very accurate observers of human nature, like Dickens, Sterne, and Fielding, whose "Tom Jones," I daresay, you may happen to know.

I could speak for hours if I were to say what influence the surgeons in London whom I have named had on my mental development. First of all, I admire the truly noble character of the profession, the good feeling of its masters to each other, their candor, their humanity in the treatment of severe cases. I can only repeat what my father said fifty years ago—the operations which I saw were, all of them, necessary, well planned, and, in most cases, executed with great dexterity. Manual dexterity was considered as a quality which scarcely deserved to be mentioned; it was only spoken of where it shone by its absence in a bungling operator. Every operation was executed with the sole view to save the patient's life or to diminish his sufferings, not to show the dexterity of a *virtuoso*. Circular amputation was preferred to the more showy flap amputation. This I had not forgotten when I had some influence in recommending the circular amputation in times of war, where it is of greater importance still than in chronic cases of civil practice. In 1827 I examined the invalids in Greenwich Hospital, on whom amputation had been performed by the flap method, and found that the fleshy cushion had disappeared entirely. Besides this, I admired English surgery for the simplicity of its application. The great conformity of principles resulting from simplicity struck me as highly valuable, because it makes a deep impression on the mind of a younger member. This conformity gives English surgery a national character. It is not the same in other countries, where only your very particular friends admit that you are right in saying that two and two make four, or that a severe gunshot fracture requires amputation.

I was well satisfied with the great caution of English surgeons in adopting innovations. I saw no resections then, and there was no trace of lithotripsy yet. It is better to begin slowly, and then to go on steadily. This is otherwise in Germany and in France, where surgeons are fond of novelties. At present you may witness the effect of greater caution. Sir Henry Thompson has eclipsed the inventor of lithotripsy, Civiale himself, whose instruments, indeed, were not worth trying till Heurteloup had found the right ones for him. Sir William Fergusson, by his articular resections, has surpassed most Continental surgeons; Mr. Spencer Wells, in ovariotomy, all living surgeons.

From what I had observed in London, I came to the conclusion that the beneficial influence of surgery and the high standing of the profession depend chiefly—(1) on the good feeling of its members towards their patients and towards each other, not excluding those of a former time; (2) on simplicity; (3) on a total abnegation of selfishness in planning and executing surgical operations.

You may ask me, gentlemen, why I could not have learned that just as well in Germany. There is no place there which can boast of such a number of great surgeons at the same time. Our greatest capitals have but a few surgeons of eminence in comparison.

Whatever may be their merit, their example is not so striking as that of a whole body acting on the same principles. In Paris the number of surgeons is greater than in our German universities of Berlin or Vienna, but not to be compared to London, which I consider is a central point of surgery for the whole globe. This, gentlemen, you may consider as the sincere opinion of a man who has watched the progress of surgery during half a century. I wish it may remain so for centuries.

After having been in London, I happened to be in Paris at a time when Lisfranc was thundering against Dupuytren, whom he used to call "le barbare de la Seine," as a sample of the good feeling amongst the profession there. It is one of the great advantages of traveling, and of seeing eminent men of other countries, that, by observing them in their activity, one may acquire a better notion of their character. Their writings excite greater interest, because we are inclined to give them greater credit. I always admired the simplicity of style in English authors in general, and of surgical writers in particular. Sterne ridicules the pompous style by mentioning the expression of his French barber about the solidity of a new wig, "You may immerse it into the ocean." An Englishman, says Sterne, would have prefered a pail of water. To avoid the barber's style, I took precious good care never to say ocean when I meant a pail of water.

After sketching these general impressions, permit me, gentlemen, to give a few particulars of the manner in which some of my English teachers have influenced me. Having so lately seen one of the greatest battle-fields of modern history—that of Sedan, where I met Mr. William MacCormac, who, from over-exertion, did not look so well as to-day—and the siege of Paris afterwards, let me speak of Mr. Guthrie first. I cannot say that I liked him personally quite so well as many of the others; but I admired his energy in maintaining the great principles acquired in the Peninsular War—the necessity of early primary operations, of tying a wounded artery, if possible, on the wounded spot itself. I have done all in my power to keep his doctrines, those of the admirable Hennen, and of old Baron Larrey, in fresh memory since 1848, when the time seemed to approach that Germany must go to war for its own development. For a man of sense, there can be no doubt about the necessity of early primary operations; but in military practice there are difficulties in which it is the duty of every medical man to maintain the sacred cause of humanity. The sentiment was appreciated even by a conqueror like Napoleon I, who said of Larrey that he was the most virtuous man he had ever known. It was one of Mr. Guthrie's best qualities that he always gave very positive reasons for what he did; so that a person could easily find out whether his own views were in accordance with Mr. Guthrie's opinions. There was no fickleness about him. I differed from him in one essential point—that of his preferring amputation for gunshot-fractured thigh to conservative treatment. Guthrie places too much stress upon the imperfections of conservative treatment, the result of which is often a very disabled limb, whose possession does not make the patient very comfortable. But these imperfections admit of improvement, while a high amputation gives no prospect of better chances; it will always remain a very dangerous operation. Our first object is to save a man's life, and the second to make him comfortable, but not in his grave.

My results of conservative treatment in gunshot-fractured thigh, during the first three campaigns of 1849, 1850, and 1866, did not go beyond 50 per cent. healed. I saw the reasons of our failures, tried to avoid them, and went on with conservative treatment. In the two campaigns of Schleswig-Holstein (1849 and 1850) the patients had to be carried considerable distances. After the battle of Langensalza, in 1866, I was unable to prevent many cases from being spoiled by an injudicious use of plaster bandages. It was in Floing, near Sedan, where we succeeded in saving 77 per cent., twenty-seven amongst thirty-five patients, who had not been carried any great distance, and were treated without putting much restraint on their shattered limbs.

From my own father I had learned the advantages of Percival Pott's position, which may be employed during the first period in most cases; in others or later the double inclined plane, or a straight wire basket, will suffice.

According to my opinion, the great principles to be followed in compound fractures in general, are—(1) dressing the wounds without lifting the limb; (2) avoiding constriction; and (3) not irritating the muscles in straining them by mechanical contrivances. A gunshot fractured thigh permits a weight to be suspended to it, keeping the limb a little at rest, like the hand of an assistant, but not an extension by weight or other contrivances, that gives the limb its proper length, except in very few cases, as mentioned by Mr. MacCormac in his "Notes and Recollections," which healed without difficulties and without any perceptible shortening. The most common case is, that for sometime after the accident the muscles retain a tendency to retract, which is increased by opposition, and ceases by-and-by in a favorable position of the broken limb. The idea of subduing muscular action by constant extension, even in compound fracture, is not new; but it had not been tried before by contrivances so dangerous as a plaster of Paris bandage. This is applied under chloroform, which relaxes the muscles; the limb is made straight, and as long as its fellow. When the action of chloroform has ceased, the muscles recover their activity, and are kept in extension in spite of their violent efforts to contract, which often break the plaster bandage. The tension, which is kept up by mechanical means, makes the sensibility rise to a high pitch, and severe inflammation follows. If the plaster bandage be loosely applied, by putting wadding and a flannel roller between, it is often well borne, but the limb is as short afterwards as if no bandage had been employed. While I was writing this in Hanover, on May 1, a young captain came to me, from whose gait no one would have thought that he had had a gunshot fractured thigh in 1870. A plaster bandage had been applied on the third day; he could not bear it. The surgeon who took it off next day told him that the fragments had taken a bad position under the bandage. From this time he was treated without restraint, and cured in six weeks, his limb lying in a wire basket. The shortening was one inch of his left lower extremity. His brother met with the same accident at the same time, but was healed with a shortening of five inches. Large splinters came away by suppuration, some of them being three inches long. The captain came to consult me about his brother. He is in service again long ago.

The danger of early plaster bandages on other parts of the skeleton is less than in the thigh; but it exists and is very great in the humerus, where pressure is very liable to stop the venous current or to drive a splinter of bone into the brachial artery. I treat these fractures by letting the arm lean on a soft cushion, which is tied to the thorax, the forearm being suspended in a sling. In Schleswig-Holstein I had twenty-four successful cases amongst twenty-nine. One of the German surgeons who took part in the late war—Dr. Rapprecht, of Munich—prefers the plaster bandage; but amongst the three cases which he had to treat there was one which proved fatal on the seventeeth day by hemorrhage, a splinter of bone having opened the brachial artery.

I differ from Mr. Guthrie, besides, in his appreciation of trephining the skull, which I have tried to exclude entirely from military practice, as useless in some and unnecessary in other cases. I consider a state of coma, from depressed skull, as no more an indication for applying the trepan than a comatose state in typhus is an indication to rouse the patient from it by any other means than those which are in accordance with his general state—cold, for instance, but not stimulants. As soon as the fragments of skull become detached by suppuration, the comatose state ceases of itself.

The greater difficulty in settling this skull question consists in this—that some patients survive the use of the trepan, or of an early extraction of splinters, and that some recover their senses very soon after the operation. This seems to be a conclusive proof of the legitimacy of active interference. But there is no depending upon it: the patient may die just as well after having recovered his senses completely, and, as experience has shown, more easily than if you let him continue comatose by not disturbing the splinters. This might have been expected, from very solid physiological reasons. By taking away the splinters at an early period, in cases where the dura mater is wounded, you open the arachnoid cavity; air and acrid matter can enter it. Brain substance, when bruised, thus becomes putrid, while it might have been eliminated by reabsorption without access of air. Subcutaneous operations practiced in modern times have done a great deal to put more stress on excluding air; but even before their time, Dease and Sir Benjamin Brodie came to a conclusion that access of air was to be avoided in cases of fractured skull, and that no interference ought to take place for depression unless it was warranted by cerebral symptoms. John Hunter had not yet arrived at this degree of caution when he said in his Lectures (Palmer's edition, vol. i., p. 493)—"All fractures of the skull may be called compound; for if not so naturally, they are made so by the removal of the scalp."

In the retrograde tendency of surgical interference with a broken skull it was an important step not to remove the scalp; but other steps were to be argued. An open scalp wound over a broken skull does not produce a great change in the danger of the case. Spreading inflammation of the membranes of the brain or deep-seated suppuration does not necessarily follow from it; but these are very likely to take place if you open the arachnoid cavity by removing the splinters which have kept it closed. When the splinters come away by a very limited suppuration at a later period, the arachnoid cavity is closed by adhesions of dura mater to the brain. It is often impossible to say, beforehand, whether the dura mater has been open or not. If it is open, the danger is rendered much greater by removing the splinters. The *Medical Times and Gazette* of 1860, contains a list of eighty-three cases in which the trepan had been used, fifty-one of whom died, and thirty-two recovered. Amongst those who did well, the dura mater had been wounded; but in three cases the others as well were such that, according to my experience, they might have recovered without using the trepan or early extraction of splinters. Gunshot fractures of the skull are always compound; their successful treatment without active interference deprives this of one of its strongholds—the presence of an open wound, which formerly seemed to permit further violence.

During the two Schleswig-Holstein campaigns of 1849 and 1850, I had to treat forty cases of gunshot-fractured skull, thirty-three of whom recovered, and seven died. We had one case of trephining with happy result, but it was of that description that it might have done well without interference. The others were subjected to an antiphlogistic treatment by ice, bleeding, purgative medicines, and low diet. The splinters were not removed before being quite loose.

I have been blamed by Mr. Pirogoff and others for totally excluding active local interference in gunshot skull fractures; many others have followed my example. You will admit, gentlemen, that there is no knowing of what use a thing may be before having tried it. My object was to know how far we might get without active interference. The result was not unsatisfactory. It was the same thing with the treating typhus patients without stimulants. By trying it on physiological principles, derived from morbid anatomy, I found it very successful.

What has pleased me most, from a medical point of view, during the late war, was to find two hospitals in Rheims and one in Versailles where the number of deaths from typhus was not above eight per cent. Weak broth and some ounces of very sour wine were all the stimulants employed till the fever was over. The wine which I tasted was so sour that it must have contained more acid than common vinegar does, as I know from comparative experiments with potash. So it probably did the same service as phosphoric acid, which I prefer, with a well-boiled water-gruel for diet during the febrile stage. The two hospitals in Rheims were close to each other; in one, the patients were cooled by immersion —in the other, by active ventilation in tents, while the results were quite the same in both. In other hospitals the mortality from typhus amounted to 25, even to 50 per cent. Nothing shows the great value of the medical art and science better than such striking differences in the results of treatment under the same circumstances in regard to constitution, causes, and symptoms.

During the greater part of my presence in London I used to see the surgical patients at St. Bartholomew's Hospital under the care of that clever and highly accomplished surgeon, Mr. W. Lawrence, whose kindness and very instructive conversation I shall never forget. I saw a great number of patients under him with phlegmonous inflammation, who were treated by incisions at an early stage before suppuration had set in. This bold practice was at that time little known in Germany, where it was spread afterwards, and is generally employed up to this day. Cases of this description do not permit hesitation, and show the use of the treatment very evidently. The effect of an antiphlogistic treatment is not so striking in many other cases. It is only by a longer experience that a surgeon is enabled to say whether a case of fractured skull, or a compound fracture of the limb, has been greatly benefited by a venesection, which has been made, not in a late period when suppuration is forming, but early, when reaction is taking place, when the face becomes flushed, and the pulse full and hard. Amongst the many wise things which Sir Astley Cooper has said, was the advice to visit a patient with a broken skull three times a day, in order to find the proper time for bleeding him. This does not produce a similar effect like an incision in phlegmonous inflammation ; it does not restore the patient's consciousness, but it keeps him alive. That this really takes place can only be judged from other cases in which bleeding at a proper time has been omitted. But bleeding is out of fashion now in Germany as well as elsewhere. The discovery that pneumonia can be cured without bleeding has been the first cause of this antipathy. I have treated pneumonia myself without bleeding, and had very good results. I lost but five patients out of 558 during ten years in the general military hospital of Hanover, from 1853 to 1864. Our patients were cupped at once, and took phosphoric acid. I never allowed this to be proof that venesection was equally unnecessary in surgical cases, which have no typical course like pneumonia. It was a mistake of former times that pneumonia might be subdued by repeated bleeding—it runs its course in spite of that. I have tried in vain to maintain its use in surgical practice; there is no swimming right across a mighty stream ; one must wait for the proper time of low water to cross it. It must be some years ago that Mr. Syme said bleeding-lancets were to be found in Great Britain no more. This, I suspect, has been the acme of antipathy to bleeding. From that moment it was no more a distinction not to bleed. Bleeding ventures to show its head again in the *Medical Times and Gazette* now rather timidly—recording cases which would have been fatal without venesection. Lancets can be easily supplied again, and a few cabbage-leaves, as Dickens says, will be sufficient to give a little practice before opening a vein in man. Perhaps I am mistaken in my expectation that bleeding will soon have its turn again. Perhaps I shall be damned in a future time for having been the last of the Mohicans recommending venesection. At all events I would in that case meet very good company—all my old friends in London.

On July 6, 1827, I witnessed the first case of hemorrhage from thrombosed vein, in St. Thomas's Hospital, under Mr. Tyrrell's care. A shoemaker had been stabbed by his own wife, with an awl, in the right upper arm. The wound appeared trifling to him —he did not notice it for some days ; then an immense swelling of the upper arm took place ; the forearm became gangrenous. Mr. Tyrrell amputated close to the axilla. In examining the separated limb, it was found that the brachial vein had been freely opened by the instrument ; that a large hole, filled with coagulated blood, had formed near the vessels. The brachial vein was thrombosed to a considerable extent above the puncture. It struck me that the internal bleeding must have taken place after the obstruction of the brachial vein, and that gangrene had been produced by stagnation. I remembered this case many years later, when I found, in military practice, that secondary hemorrhages in open wounds take place from similar causes. I described them under the name of phlebostatic hemorrhages. Others prefer to call them pyæmic, putting little stress upon the venous obstruction. But pyæmia does not always exist in these cases, and the influence of venous obstruction is evident. You will see this if you should happen to bleed again, which must be done by stopping the venous current above the place where you open the vein. Every open wound would bleed under a similar contrivance. Let me observe here, what I have forgotten to mention elsewhere, that capillary thrombosis, of some extent, below an injured vessel must have the same effect as thrombosis of the main vein, because it increases the degree of pressure which the column of blood exerts in entering the limb. The quality of the blood in it must become altered by impediments of either kind, and healthy nutrition cannot be kept up.

These observations are of great interest in military surgery: they teach us to be very cautious in treating wounds which may have affected vessels of considerable size, whose bleeding has been arrested by bruising or by coagula. The injured vessels may heal without hemorrhage, if the reflux of venous blood remain free; but if there be any obstruction, either by capillary or venous thrombosis, secondary hemorrhage can occur. Before this takes place the wound often changes its aspect from altered nutrition. Mr. Guthrie's plan of tying a wounded artery on the spot often does very well in minor vessels, but it often fails in the femoral artery. The large vein accompanying it has often been torn or bruised by the same ball. After tying the artery on the spot, the vein often becomes totally inpermeable, and then hemorrhage recurs, or the limb becomes gangrenous. It may be proper in some cases to gain time by putting a ligature above the wounded spot; before new hemorrhage occurs, the vein may have undergone a favorable change. In other cases, it is better to amputate at once.

I cannot dismiss Mr. Tyrrell's name here without mentioning how I used to admire his cataract operations. He did them generally by a superior corneal incision of the greatest regularity. I adopted his method of sitting behind the patient's head in operating on the right eye. I had seen Graefe, the elder, in Berlin; Jaeger, the elder, in Vienna, and Roux in Paris, perform extraction as well with the left as with the right hand, but I preferred the more cautious English way. I often thought of Tyrrell's beautiful operations and their results when the time came that iridectomy seemed necessary for the great majority of cataracts before extracting them. I have hailed Dr. Liebreich's innovation as a candid acknowledgment that modern oculists had gone too far in this respect, and that the iris ought to be spared if possible or reasonable.

To Sir Benjamin Brodie I feel very thankful to this day for what I have seen of him in treating diseases of the urinary organs, but chiefly for his skill in diseased joints. He was the first surgeon who enjoined the doctrine of keeping diseased joints at rest by putting them on a splint. The leather splints which he recommended had been partially superseded by starch or plaster bandages, but I use the leather splints constantly in my chronic cases, when the patient is to go out to bathe, or to use other local applications. Sir Benjamin Brodie's influence on the treatment of articular diseases has been great in Germany. His work on the subject was translated, in 1821, by Dr. Holscher, of Hanover. It had to fight its way in Germany against Rost's authority, who had introduced the red-hot iron as a general remedy for most chronic cases. It is used no more now. The great principle which Mr. Hilton has so ably advocated, of keeping diseased parts at rest, either by mechanical or by physiological means, has done away with the red-hot iron. It was Sir Benjamin Brodie's farther merit to point out cases where articular disease is spurious, and where rest proves hurtful. I consider Brodie's work on local nervous affections, published in 1837, as one of the greatest value on account of the number of persons who may be benefited by his doctrines. I had given a short extract of it in my "Manual of Surgery," but this had no effect in rousing the public attention. My German countrymen imagined that local nervous affections were a particular gift of nature to English young ladies. Not a month passes but I see a striking case in Hanover. My son-in-law, Professor Esmarch, who travels a great deal during his vacations, has been able to pick out a number of cases in different parts of Germany, and to extricate them from the spider's web of injudicious treatment. Perhaps I ought to have written myself about this subject more at large, but I despaired of doing it better than Sir Benjamin Brodie. Professor Esmarch published a small volume last year on "Articular Neurosis," which, I hope, will go far in spreading Sir Benjamin Brodie's doctrines, whose German translation has failed to produce the desired effect. This subject is intimately connected with the exertions of that great genius, whose discovery of the different roots of motor and sensitive nerves has spread a lustre on our century.

Perhaps no man has given so much to think of to his contemporaries as Sir Charles Bell. He did not live long enough to witness the wide expanse of studies derived from so simple a source as that of the different roots. Bell's researches on paralysis of the facial nerve alone were sufficient to create a number of similar ones. The connection of this illness with rheumatism; the liability which persons have for it who are subject to abdominal affections; to impediments in the circulation of the abdominal viscera, pointed to other diseases with diminished nervous energy—in the organs of sight and of hearing, for instance.

In following the hints given by Sir Charles Bell, I found that every local affection originating from violence, or spontaneous inflammation, was influenced in its course by an enlarged liver or spleen, and that for a cure it is necessary to reduce their size. This accounts for the very general, but more empirical, use of blue pill and bark, and advises us to examine the size of the liver and spleen by percussion, especially in chronic cases which show very little tendency to heal—in secondary or tertiary syphilis, for instance.

Marshall Hall's discovery of the reflex function gave a new stimulus to thought on the great importance of Bell's discovery, from which it had taken its origin. This doctrine of the reflex action of the nervous system can only be compared to the discovery of the blood circulation. It gave an idea of the manner in which the nervous action is kept on day and night, and reflex action following the other. Trying to understand this action, I was led to assume a second principle, which is ultimately blended with reflex action. I found that immoderate reflex action in muscles, or muscular organs, was generally combined with painful feelings, sometimes in the neighborhood of, sometimes remote from, the seat of spasm. As a similar train of combined sensations must take place during healthy action of the muscles or muscular organs, I guessed that the action of the muscular system was necessary to maintain the nervous energy.

Sir Benjamin Brodie, in his work on local nervous affections, opposes the popular use of the name of spasm for painful feelings. This is the case in Germany. People make no difference between *Krampfe*, spasm, croup, and *Schmerz*, pain. There is some reason for it; where there is spasm there used to be pain, but often in remote parts. Instead of pain, spasm is often combined with altered sensibility, partial or general. The German name for hysteria is "mutterkrampfe," mother cramps. The uterus being a muscular organ, it may well be that hysteria partially consists of habitual spasm of the uterus, as a reflex action from the ovaries, or from other parts of the system. This idea was well known in former times. You can find it in Shakespeare's "King Lear," who is made to say, "How this mother swells up towards my heart."

One of the most striking examples of pain originating in spasm, is that of the glans penis, from contractions of the bladder around a stone in it. Another well-known example is pain in the knee, arising from reflex spasm of the flexor muscles of the hip-joint, from inflammation of its bones, sometimes from other causes.

In a particular case, where there was no inflamed hip-joint, I succeeded in doing away with the knee-pain by dividing the rectus and pectineus muscles. I have pointed out sensations, combined with muscular action, in all the organs of sense and in many cases of disease. I have written on my theory of combined motor and sensitive nervous energy; first, in Hanover, 1837, in the *Gottenger Gelehrten Anzeigen*, an article which I reproduced in Latin, after having become Professor of Surgery in Erlangen. I have followed up this subject no farther, because it would have cost me the exertion of a whole life to carry it to a degree of perfection equivalent to a clear demonstration by physical evidence. But the fact that every pain, which evidently does not depend upon local alteration of texture, depends on spasmodic action of some muscular organ, has been of great use to me in practice, and I can advise you to put this question to yourself in every case of that description—where is the seat of spasm? Remember that it is as well in the voluntary as in the involuntary ones that spasms can take place. Perhaps you little suspect that the liver is an organ whose muscular energy is of great importance. But if you had felt the pain in passing a biliary calculus, you would think otherwise. I have felt it myself, and took six grains of opium in one night for it.

Pains in remote parts of the body are very frequent in liver complaints, in the shoulder, in the head, or in other parts. I could tell a great number of cases in which local nervous affection of an extremity proceeded from the liver, others from the accumulation of hard fæces in the large intestines, keeping up spastic action for their expulsion. It is very easy to cure them, after having found the real cause, without any topical application whatever to the affected limb.

After having spoken of such a variety of things and for such a length of time already, let me bid you farewell now, gentlemen, and thank you heartily for the kind attention with which you have been listening to me. I wish you may remember your studies in London with the same feelings of gratitude and satisfaction as I do after forty-four years of practical life.—*Medical Times and Gazette.*

---

*Mud Baths.*

There are some baths in Hungary and Bohemia where visitors plunge themselves into a basin of soft mud instead of crystal water. These mud baths, *moorbader*, are quite popular in their neighborhood. So at Saint Amand, in the north of France, there are some famous mud baths, salutary for diseased articulations, rheumatism, atrophy, etc. The patient is planted up to his neck in a hole in the soil filled with a black, soft mud, warmed by the thermal waters which well up through it.

We have some also in this country. In a late paper we notice that Grace Greenwood has become a convert to mud baths, and chants the praises of the Indian baths at the Geyser Springs, a California retreat, at the respectable altitude of 1690 feet above the sea. It is said that Edwin Forest, while suffering from a severe attack of rheumatism some years ago, went into the Indian mud bath one day, and came out all ready to play Othello or Metamora. —*Med. Reporter.*

The baths at Hot Springs, Arkansas, are said to have been much more efficient, formerly, when taken in a similar manner.—Ed.

